# The English Conjoint Course

**Published:** 1909-09-04

**Authors:** 


### THE ENGLISH CONJOINT COURSE.

The student who is desirous of taking the Conjoint qualification—that is to say, the double diploma of Member of the Royal College of Surgeons of England and Licentiate of the Royal College of Physicians of London—has to comply with the regulations laid down for medical qualifications generally. When registered as a medical student he has to show that he has attended lectures and practical work in chemistry and biology before he is allowed to proceed to either the first or the second part of his "first." If he wishes to take the third part at the same time he has to show proof that he has been properly instructed in practical pharmacy by a recognised chemist or registered practitioner. The subjects for the first examination are chemistry and chemical physics, biology, and pharmacy. In the first part the examination is partly written and partly practical; in the second and third parts it is wholly oral. The practical part consists of simple quantitative and qualitative analysis, and candidates usually find the test a comparatively easy one. Assuming that the student has had some preliminary training, such as can usually be obtained at the general schools from which he passes his preliminary, he ought not to take a longer time than six months to pass the first and second parts of the "first." Many students take both parts after only three months' work.

The biology required for the first examination is exceedingly elementary. The examination is entirely oral, lasting, like all the *viva voce* tests of the first and second examinations, for a quarter of an hour. The candidate is asked to "spot" certain parts, to identify specimens under the microscope or in jars, and to answer questions connected with the subject he has been working at. The scope of the examination is shown by a perusal of the standard text-book, "Chalmers Mitchell's Biology," which is the only book the student need obtain, so far as his work in this part of the first is concerned.

For the chemistry there are many good text-books. Those most generally used are "Corbin and Stewart's Chemistry" and "Luff and Page's Chemistry" (Lewis, 7s. 6d.), of which the former is specially intended for Conjoint students, while the latter contains more information, especially on the chemistry of alkaloids.

The third part of the first examination—practical pharmacy—the student may with advantage defer till he has acquired some knowledge of drugs in his ward work. This part is purely oral, the candidate being asked to "spot" drugs, to name their doses, preparations and strengths, and sometimes he is questioned on their active principles.

Having passed his first, or the first two parts of it, the student is allowed to enter the dissecting room and commence his study of anatomy and physiology. He must be engaged in the study of these two sciences during one whole summer and one whole winter session before he can enter for his second examination, and during that time he must have actually dissected the several "parts," and must have attended a course of instruction in histology and practical physiology, together with a course of lectures (six months) in physiology and anatomy at a recognised medical school.

The second examination is both oral and written. There are two papers, one in each subject, and each has six questions, of which the candidate must answer at least four within the stipulated three hours. Here, too, as in the "first," the questions are straightforward, and the student who has displayed average diligence in his studies should not have much difficulty in satisfying the examiners at the end of his second year. The *viva voces* are purely objective, and the candidate is rarely asked to describe any structure.

In anatomy Conjoint men will do well to stick to one text-book of general and one handbook of practical anatomy. Individual taste and inclination will guide the selection of a text-book in the majority of cases, for there are so many excellent volumes to be had that selection between them, on their merits, is impossible. Those most commonly used are Gray's "Anatomy" (Longmans, 32s.), Cunningham's "General Anatomy" (Oxford Publications), and Morris's "Anatomy"; and Cunningham's "Manual of Practical Anatomy" (Oxford Publications, 2 vols., 12s. 6d. each). In physiology the more commonly used text-books, such as Halliburton's "Physiology" (Murray, 18s. 6d.), Starling's "Physiology" (Churchill, 15s. 6d.), Schäfer's "Histology" (Longmans, 9s.), Halliburton's "Chemical Physiology" (Longmans, 4s. 6d.), and Hill's "Practical Physiology" (Arnold, 12s. 6d.), will generally suffice.

For the final or qualifying examination, the student must produce evidence of having attended at a recognised medical school specified courses of lectures on medicine, surgery, midwifery, pathology (including pathological histology and bacteriology), pharmacology and therapeutics, forensic medicine and insanity, and public health; of having received systematic practical instruction in these subjects; and of having performed operations on the dead body. In addition he must show that he has attended, since passing his second examination, the practice of medicine and surgery, demonstrations in the post-mortem room, clinical lectures on medicine and surgery and diseases of women, and of having served as clerk and dresser in the wards. He must also have obtained a certificate of instruction and proficiency in the administration of anæsthetics, and have attended special classes in ophthalmology, in fevers, in lunacy, and in vaccination, and must have attended twenty labours. In addition he must be twenty-one years of age or older.

The examination is in three parts, medicine, surgery, and midwifery, and the student is allowed to take the third part at the completion of four years of medical study. The examination in each part comprises a paper of six questions, four of which must be dealt with, and a *viva voce*. In surgery and medicine there are additional *viva voces* in anatomy and pathology, together with a long *viva* on "clinical cases."

